# MicroRNA-21 Promotes Cell Growth and Migration by Targeting Programmed Cell Death 4 Gene in Kazakh's Esophageal Squamous Cell Carcinoma

**DOI:** 10.1155/2014/232837

**Published:** 2014-10-21

**Authors:** Tao Liu, Qing Liu, Shutao Zheng, Xiangpeng Gao, Mang Lu, Chenchen Yang, Fang Dai, Ilyar Sheyhidin, Xiaomei Lu

**Affiliations:** ^1^Clinical Medical Research Institute, First Affiliated Hospital of Xinjiang Medical University, Xinjiang Uygur Autonomous Region, Urumqi 830054, China; ^2^State Key Lab Incubation Base of Xinjiang Major Diseases Research, First Affiliated Hospital of Xinjiang Medical University, Xinjiang Uygur Autonomous Region, Urumqi 830054, China

## Abstract

Esophageal cancer (EC) is the eighth most common cancer worldwide and the sixth most common cause of cancer death. There are two main types of EC—squamous cell carcinoma (ESCC) and adenocarcinoma (EAC). Although some advances in the exploration of its possible etiological mechanism were made recently including behaviors and environmental risk factors as well as gene alterations, the molecular mechanism underlying ESCC carcinogenesis and progression remains poorly understood. It has been reported that miR-21 was upregulated in most malignant cancers, the proposed mechanism of which was through suppressing expression of programmed cell death 4 (PDCD4). In present study, it is firstly reported that miR-21 was upregulated in Kazakh's ESCC and that miR-21 played a negative role in regulating PDCD4 using in situ hybridization (ISH) and luciferase reporter approach. Morever, in model of ESCC xenografted nude mice, miR-21 maybe used as an effective target in the treatment. The present results demonstrated that miR-21 may be a potential therapeutic target in management of ESCC.

## 1. Introduction

EC is the eighth most common malignancy and the sixth leading cause of cancer death in the world [1]. ESCC was the most frequent type of histopathology in China, whereas EAC is the more prevalent in western countries [2]. Xinjiang, the northwestern part of China, where the Kazakh's ESCC has been reported to be abnormally higher than other minorities [3]. Although some advances in the exploration of its possible etiological mechanism were made recently including behaviours and environmental risk factors as well as gene alterations [3, 4], the complicated mechanism which the Kazakh ethnics abnormally suffered the highest is still largely unknown. On the other hand, in spite of improvement in the treatment for EC, the associated mortality of EC remains disappointedly lower, with its 5-year survival rate less than 20% [1]. Thus, it is desperately needed to understand the mechanism of EC.

MicroRNAs (miRs) are short noncoding RNAs which control gene expression by targeting specific genes in a posttranscriptional way [5, 6]. Being found to play an important role in regulation of fundamental cellular processes, including proliferation, migration, and differentiation, they have been shown to be involved in the pathogenesis of tumors acting as oncogenes or tumor suppressor genes [7–10]. Of all the cancer-related miRs, miR-21 has been invariably and consistently found to be overexpressed in almost every diverse type of malignant tumors [11–18] and reported to be mediated in cancer-related process. Recently, several target genes of miR-21 have been identified including the phosphatase and tensin homologue (PTEN) [12], tropomysin 1 [13], the programmed cell death 4 (PDCD4) [14], and maspin [15]; all of these proposed target genes have miR-21 binding site by bioinformatic analysis. Despite the relevant report regarding miR-21 and its downstream target PDCD4 has been published in Janpanese patients with ESCC. The present study firstly indicated the key data about regulation of miR-21 over PDCD4 in Kazakh's ESCC, experimentally demonstrating that targeting against miR-21 could be a potential therapeutic strategy in the management of patients with ESCC.

## 2. Materials and Methods

### 2.1. Tissues and Cell Line

Pairs of primary ESCC and adjacent normal tissues were obtained from 50 patients, who were hospitalized from 2007 to 2008 at the First Affiliated Hospital, Xinjiang Medical University, China. The present study was approved by the local Medical Ethics Committee and signed informed consent was obtained. None of the recruited patients received treatment before surgery. All tissues were formalin-fixed and paraffin-embedded (FFPE) for pathological diagnosis. Eca109 cell line was purchased from WuHan University (Hubei; WuHan).

### 2.2. Cell Culture and Transfections

Eca109 cells were maintained in DMEM (Gibco) supplemented with 10% FBS (Gibco) in a 5% CO_2_ humidified incubator at 37°C and transfected with miR-21 mimics, miR-21 inhibitor, and scramble sequence by Lipofectamine 2000 in Eca109 cells as described previously [16].

### 2.3. Luciferase Reporter Assay

Eca109 cells were transfected with two luciferase reporter vectors using Lipofectamine 2000, each of which contains full length of pre-miR-21 sequence and 3′-UTR of PDCD4, respectively (Origene). Luciferase reporter vector with PDCD4 mutant target was transfected in parallel as control. Luciferase activities in the cells were assayed using a luciferase assay kit (Promega).

### 2.4. Wound-Healing Assay

As Eca109 cells were grown to 85% confluence in 6-well plates, a wound was incised with a sterile 10 uL pipette tip, in the center of the dishes, to create extended and definite scratches with a bright and clear field. Phosphate-buffered saline (PBS) was used to remove the detached cells by washing the cells once. After transfection with miR-21 mimics, inhibitor, and scramble sequence for 24 h, 48 h, and 72 h, respectively, images of cellular morphology from different groups and migratory cells images from the scratched boundary were observed and acquired under the light microscope. The distance between the two sides of the wound was measured with a graduated ruler and relative scratch breadth was determined by a ratio of average breadth in treatment cells versus the average breadth in control cells.

### 2.5. ISH and Immunohistochemistry (IHC)

Five-micrometer-thin sections were cut from the FFPE blocks. Expression of miR-21 was detected by ISH with probes for miR-21 according to manufacturer's protocol of microRNA ISH Optimization Kit (Exiqon). The slides of IHC were dewaxed and rehydrated in a gradient of ethanol and xylene. The antigen retrieval step consisted of microwaving sections in 0.01 M citrate buffer at pH 6.0 for 20 min in 800 W microwave oven operated at full power. The sections were then allowed to cool at room temperature (RT), and then it was treated with 0.3% H_2_O_2_ in methanol for 15 min at RT. After washing three times with PBS, they were incubated with mouse monoclonal antibody against PDCD4 (Cell Signaling Technology) from human source.

### 2.6. Tumor Xenografts

To evaluate* in vivo* tumorigenesis, ESCC xenografting mouse model was established. Male BALB/c-nu mice of 4 weeks of age were prepared for ESCC implantation. All animals were maintained in a sterile environment on a daily 12 h light/12 h dark cycle. After resuspension in PBS, Eca109 cells (5 × 10^6^/mouse) were injected subcutaneously into the flanks of the nude mice. One week after implantation when the tumor became visually palpable at the size of 2 mm in diameter, intratumor injection with 50 *μ*g of miR-21 inhibitor dissolved in 100 *μ*L of DMEM mixed with 3 *μ*L of Lipofectamine 2000 (Invitrogen) was performed twice a week. Tumor volume (TV) was calculated weekly for 4 weeks according to the formula, TV (mm^3^) = length × width^2^ × 0.5. Tumor xenografts were harvested, weighted, and snap-frozen.

### 2.7. Statistics

All statistical analysis was carried out using SPSS for Windows version 13.0 (SPSS). Student's *t*-test and one-way ANOVA were used to analyze the relationship between miR-21 expression and clinicopathologic characteristics. *χ* ^2^ test was applied to analyze the relationship between PDCD4 expression and clinicopathologic features. To measure the association between pairs of variables, Spearman order correlations were run. Kaplan-Meier survival curves were plotted and log-rank test was done. The significance of various variables for survival was analyzed by Cox proportional hazards model in a multivariate analysis. Results were expressed as mean ± SD. *P* values less than 0.05 in all cases were considered statistically significant.

## 3. Results

### 3.1. PDCD4 Was a Direct Target of miR-21

**Figure 1:**
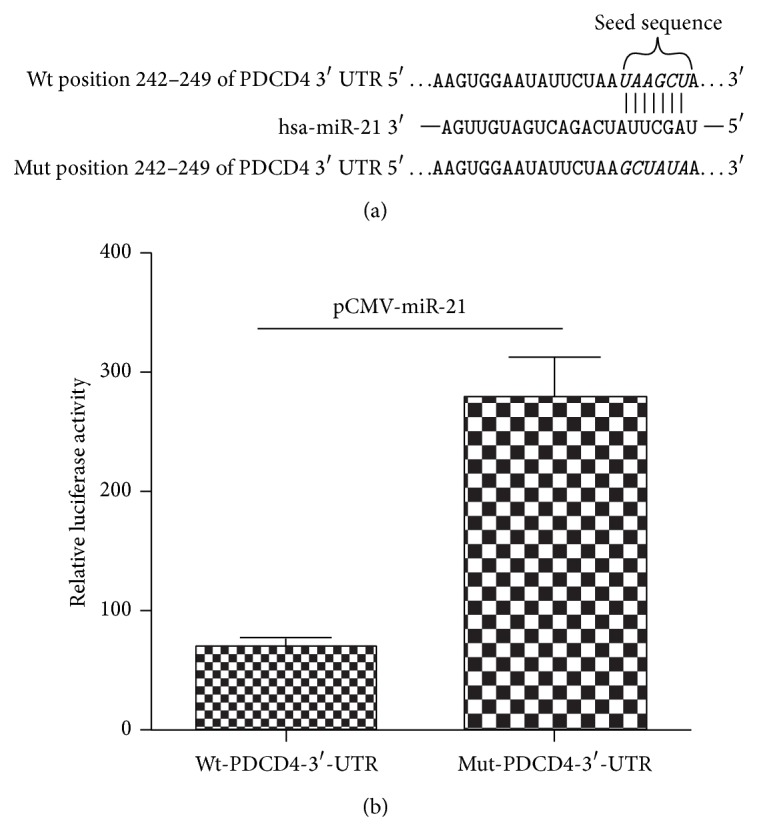
miR-21 directly regulated PDCD4-3′-UTR. (a) Prediction result of targetscan (http://www.targetscan.org/): PDCD4-3′-UTR-containing reporter constructs of miR-21. (b) Reporter assay, the cells were transfected with wild reporter plasmid or mutation reporter plasmid and miR-21 plasmid.

To determine whether PDCD4 protein was one of the downstream targets of miR-21, Eca109 cells were transfected simultaneously with two different luciferase reporter vectors with full length of 3′-UTR of PDCD4 and pre-miR-21 sequence, respectively, as described previously [14]. The transient transfection Eca109 cells with the reporter plasmid containing pre-miRNA-21 sequence led to a significant increase of luciferase activity of PDCD4 reporter activity in comparison with the negative control. However, the activity of the reporter construct deleted at the seed sequences of miR-21 target site was unaffected by a simultaneous transfection with mutagenesis of mature miR-21 seed sequence. On the other hand, to confirm the observation using luciferase reporter assay, the prediction of whether miR-21 complements PDCD4 protein sequence, targetscan website was used for theoretical verification. It can be showed clearly that 3′-UTR of PDCD4 was the direct target of miR-21 (Figure 1).

### 3.2. miR-21 Promoted Cell Migration

**Figure 2:**
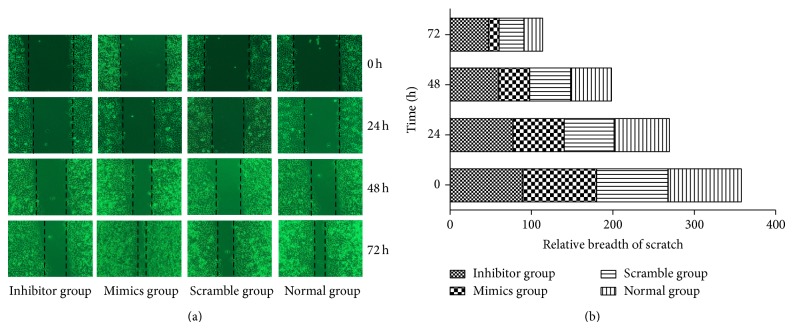
miR-21 promoted tumor cell migration after transfection. (a) Cell migration ability was assessed using wound-healing assay. Eca109 cells were transfected with miR-21 mimics, inhibitor, and scrambled sequence and were assessed for migration by wound-healing assay at 0 h, 24 h, 48 h, and 72 h after transfection. Images of migratory cells from the scratched boundary were observed and acquired with light microscope (10 × 10). Similar results were obtained in three independent experiments, and shown were representative figures. (b) Statistical analysis of wound-healing. There was significant difference between miR-21 mimics and control group (^*^ *P* < 0.05).

Wound-healing assay showed that miR-21 could dramatically promote the mobility of Eca109 cells after exogenous upregulation (Figure 2). Eca109 cells were transfected with miR-21 mimics, inhibitor, and scrambled sequence and were assessed for migration by wound-healing assay at 0 h, 24 h, 48 h, and 72 h after transfection. Images of migratory cells from the scratched boundary were observed and acquired with light microscope (magnification 10 × 10). Similar results were obtained in three independent experiments, and shown was representative Figure 2(a).

In statistical analysis of wound-healing (Figure 2(b)), there was significant difference between miR-21 mimics and control group (^*^ *P* < 0.05).

### 3.3. miR-21 Was Overexpressed in ESCC Compared with Paired Adjacent Normal Tissues

**Figure 3:**
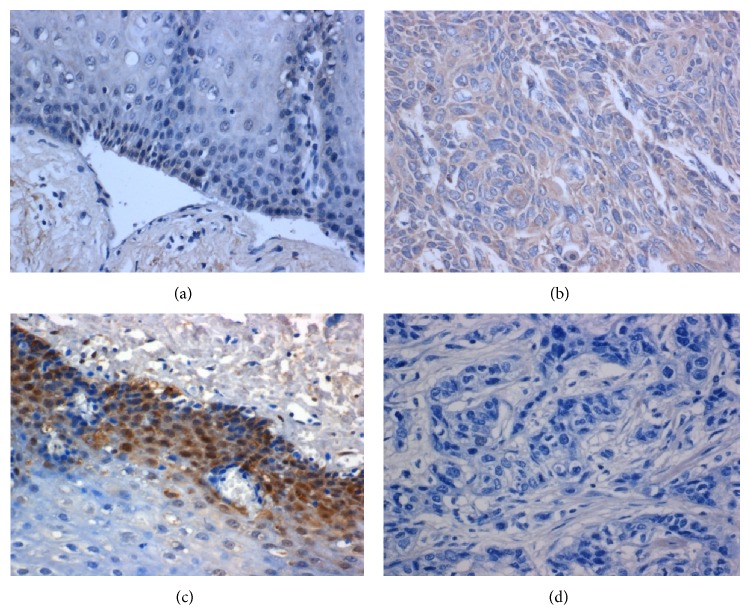
miR-21 negatively correlated with PDCD4 expression in ESCC tissues. ((a), (b)) miR-21 was detected as a weak positive staining in normal epithelium, strongly positive staining of the tumor. ((c), (d)) PDCD4 was detected as a strongly positive staining in normal epithelium, weak positive staining of the tumor.

To determine and visualize the expression status of miR-21 in Kazakh's ESCC and paired control, ISH was employed for its advantages in localization and qualitative expression in FFPE blocks in comparison with qRT-PCR techniques, which was adopted as classical approach in the detection of miRNAs. There were 50 paired Kazakh's ESCC tissues and matched adjacent normal controls were enrolled. MiR-21 was extremely significantly overexpressed in Kazakh's ESCC tissues in comparison with paired adjacent normal control (*P* < 0.001) (Table 1). In normal squamous epithelium, there was slightly positive staining for miR-21, whereas ESCC showed strongly positive staining (Figures 3(a) and 3(b)). The scramble sequence probe showed no significant staining in either ESCC or matched adjacent normal tissues.

### 3.4. PDCD4 Expression Correlated with Clinic-Pathological Features

To further confirm the upregulation and the localization of PDCD4 in Kazakh's ESCC and paired adjacent normal tissues, the rabbit anti-PDCD4 antibody was used for IHC in enlarged 50 pairs of tissues. Significantly low expression of PDCD4 protein was displayed in ESCC compared with matched tissues. Positive immunoreactivity of PDCD4 was located in cell nucleus. Most of normal esophagus epithelium showed high expression of PDCD4 whereas low expression was detected in ESCC (Figures 3(a) and 3(b); Table 1). The following data was analysed between PDCD4 expression and clinical pathologic parameters. Among all clinicopathological parameters available, including gender, age, differentiation, and lymph node metastasis, only the tendency of cell differentiation tended to be obviously associated with PDCD4 expression, but no significance was observed (*P* = 0.056).

### 3.5. miR-21 Was Conversely Correlated with PDCD4

PDCD4 protein, a putative downstream target of miR-21, has been proposed several times in previous studies. The expression of PDCD4 was determined using IHC with necessary controls in the same panel of FFPE blocks as detected for miR-21. It was shown that PDCD4 was significantly lower expressed in ESCC compared with paired adjacent normal controls, the trend of which was quite opposite with that of miR-21. Clinicopathological analysis showed that PDCD4 protein correlates inversely with miR-21 expression (*P* < 0.05) (Table 2).

### 3.6. Prognostic Analysis of miR-21 and PDCD4 Expression

**Figure 4:**
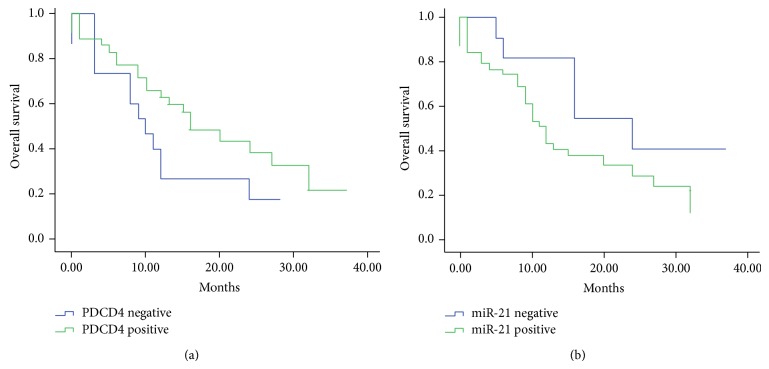
Prognostic analysis of miR-21 and PDCD4 expression. 50 ESCC patients with their following information available were classified into negative expression group and positive expression group. Shown are Kaplan-Meier overall survival curves of two group patients; log-rank test was used. (a) Expression of PDCD4 protein is associated with overall survival of ESCC patients. (b) Expression of miR-21 is associated with overall survival of ESCC patients.

To investigate the prognostic significance of miR-21 and PDCD4 expression in Kazakh's ESCC, Kaplan-Meier analysis was performed illustrating that there is no significant correlation between poorer overall survival and disease-free survival in the ESCC case. But the tendency of survival status tended to be significantly associated with miR-21 and PDCD4 expression (Figure 4).

### 3.7. miR-21 Inhibitor Suppressed Tumorigenesis of ESCC Xenografts

**Figure 5:**
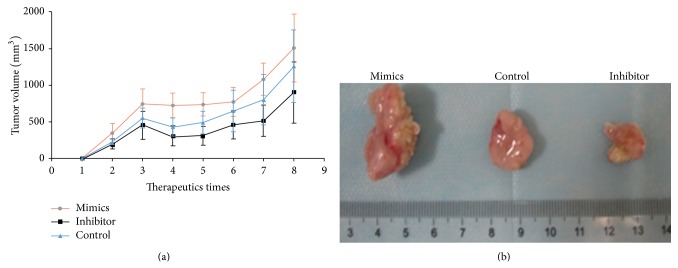
miR-21 promotes tumorigenesis of ESCC in xenografted nude mice. Twice per week intratumor injection of miR-21 mimics, inhibitor, and scramble reduces tumor volume followed for 28 d after implantation. (a) Evaluation of tumor size at different therapeutic times; (b) gross morphology of tumors resected from different groups.

As miR-21 inhibitor can prevent ESCC cell growth* in vitro* [16], to verify whether or not miR-21 inhibitor could suppress the tumor formation* in vivo*, nude mice xenografted with Eca109 cells were employed. Biweekly intratumor injection with miR-21 inhibitor, and with scramble sequence as control, tremendously reduced tumor size and tumor weight. It showed that the tumor volume was significantly lower in the group treated with miR-21 inhibitor than others (Figure 5), suggesting that miR-21 could be used as potential therapeutic target in the treatment of ESCC.

## 4. Discussion

In present study, miR-21 was significantly upregulated in Kazakh's ESCC tissues compared with paired adjacent normal controls, and that miR-21 directly negative-regulated PDCD4 protein and that miR-21 could be used as potential therapeutic target in the treatment of ESCC.

With regard to the interplay between miR-21 and PDCD4, which was definitely verified by a series of similar studies, that miR-21 was generally overexpressed in cancerous tissues versus paired normal controls, regardless of its tumor types or origins [12–16]. However, clinicopathological analysis showed that there was no significant difference between expression of miR-21 and gender, age, differentiation, and lymph nodes metastasis (*P* > 0.05), which was inconsistent with Ren et al. previous report in tongue squamous cell carcinoma [16]. The possible reason for the analytic discrepancy may be due to the fact that the detection approach was different. In Ren et al.'s study, they used qRT-PCR rather than ISH. On the other hand, the clinical sample types were also different. Fresh snap-frozen tissues were employed in their study, whereas FFPE blocks were recruited in our study. Similar studies also include another earlier report about miR-21 expression in ESCC; Hiyoshi et al. [14] also used qRT-PCR technique to detect the status of miR-21 in fresh ESCC tissues and paired controls, and as further confirmation, ISH approach was performed on FFPE blocks; it could localize gene expression to specific cell types in specific regions. They have consistently reported that miR-21 expression trend was highly consistent no matter what the approaches used; that is, miR-21 was extremely significantly overexpressed in ESCC tissues compared with paired normal controls, and that miR-21 expression was extremely significantly correlated with venous invasion and lymph nodes metastasis.

As regards the inverse relationship between miR-21 and PDCD4 expression, our results were consistent with Asangani et al.'s [17] previous claim that miR-21 was conversely correlated with PDCD4 using a large panel of colorectal cell lines, as many as 10 different colorectal cell lines. However, they have failed to provide data concerning inverse relation between miR-21 and PDCD4 using clinical tissues. Asangani and colleagues originally reported that miR-21 posttranscriptionally downregulates PDCD4, as measured in as many as ten different colorectal cancer cell lines. Subsequently, Hiyoshi et al. and Ren et al. [14, 16] confirmatively found the phenomenon firstly described by Asangani in ESCC and tongue squamous cell carcinoma both* in vitro* and* in vivo,* respectively, and independently. Moreover, prognostic analysis of both PDCD4 and miR-21 expression showed that neither of PDCD4 nor miR-21 can be used as independent prognostic factor in our study, which is inconsistent with Ren et al.'s conclusion that miR-21 expression was an independent prognostic factor indicating poor survival and which is consistent with Cao et al.'s [18] results that PDCD4 was not an independent prognostic factor in gastric cancer with 187 cases of patients; the possible reason contributing to the discrepancy may be that samples enrolled to be insufficient to arrive at statistical significance. Despite the limited number of ESCC cell line and clinical samples employed in our study, we have successfully and experimentally replicated that miR-21 promotes tumour growth and migration in ESCC through directly repressing PDCD4.

Taken together, our results in ESCC xenografts imply that target miR-21 may hold great promise for designing novel therapeutic strategies against ESCC carcinogenesis and development. MiRNA AMO or mimics could potentially be used as single therapeutic agents or in combination with other conventional chemotherapies or radiotherapies to achieve optimal therapeutic effect.

Finally, the present study demonstrated that miR-21 promoted proliferation, migration of ESCC both* in vitro* and* in vivo*, playing an important role in the carcinogenesis of ESCC. Thus, miR-21 maybe serves as potential target for the treatment of ESCC.

## Figures and Tables

**Table 1:** The expression of miR-21 and PDCD4 in paraffin-embeded ESCC and matched tissues by ISH and IHC.

Sample (*n* = 50)	miR-21 expression		*P* value	PDCD4 expression		*P* value
	Negative (−)	Positive (+)		Negative (−)	Positive (+)	
ESCC	13 (26.4%)	37 (73.6%)	0.004	35 (70.0%)	15 (30.0%)	0.000
Normal	27 (51.0%)	23 (49.0%)		12 (24.0%)	38 (76.0%)	

**Table 2:** Correlation between expression level of miR-21 and PDCD4 in 50 pairs of ESCC.

	PDCD4 expression		*χ* ^2^	*P* value	*R*
	Negative (−)	Positive (+)			
*ESCC *					
miR-21 expression					
Negative (−)	3 (6.0%)	10 (20.0%)	7.144	0.010	−0.371
Positive (+)	32 (64.0%)	4 (8.0%)			

*Normal *					
miR-21 expression					
Negative (−)	3 (6.0%)	24 (48.0%)	5.346	0.020	−0.327
Positive (+)	9 (18.0%)	14 (28.0%)			
